# Effect of postharvest UV-C treatment on the bacterial diversity of Ataulfo mangoes by PCR-DGGE, survival of *E. coli* and antimicrobial activity

**DOI:** 10.3389/fmicb.2013.00134

**Published:** 2013-06-05

**Authors:** Rocío Fernández-Suárez, Guadalupe Ramírez-Villatoro, Gloria Díaz-Ruiz, Carlos Eslava, Montserrat Calderón, Arturo Navarro-Ocaña, Andrea Trejo-Márquez, Carmen Wacher

**Affiliations:** ^1^Lab 324, Conjunto E, Departamento de Alimentos y Biotecnología, Facultad de Química, Universidad Nacional Autónoma de MéxicoMexico City, Mexico; ^2^Departamento de Salud Pública, Facultad de Medicina, Universidad Nacional Autónoma de MéxicoMexico City, Mexico; ^3^Ingeniería Química, Biotecnología de Alimentos, Instituto Tecnológico de TepicTepic, Mexico; ^4^Laboratorio de Postcosecha de Productos Vegetales, Facultad de Estudios Superiores Cuautitlán, Centro de Asimilación Tecnológica, Universidad Nacional Autónoma de MéxicoMexico City, Mexico

**Keywords:** PCR-DGGE, mangoes, UV-C treatment, bacterial communities, food safety, *E. coli*, antimicrobial activity, phenolic compounds

## Abstract

Since Mexico is the second largest exporter of mangoes, its safety assurance is essential. Research in microbial ecology and knowledge of complex interactions among microbes must be better understood to achieve maximal control of pathogens. Therefore, we investigated the effect of UV-C treatments on bacterial diversity of the Ataulfo mangoes surface using PCR-DGGE analysis of variable region V3 of 16S rRNA genes, and the survival of *E. coli*, by plate counting. The UV-C irradiation reduced the microbial load on the surface of mangoes immediately after treatment and the structure of bacterial communities was modified during storage. We identified the key members of the bacterial communities on the surface of fruits, predominating *Enterobacter* genus. Genera as *Lactococcus* and *Pantoea* were only detected on the surface of non-treated (control) mangoes. This could indicate that these genera were affected by the UV-C treatment. On the other hand, the treatment did not have a significant effect on survival of *E. coli*. However, genera that have been recognized as antagonists against foodborne pathogens were identified in the bands patterns. Also, phenolic compounds were determined by HPLC and antimicrobial activity was assayed according to the agar diffusion method. The main phenolic compounds were chlorogenic, gallic, and caffeic acids. Mango peel methanol extracts (UV-C treated and control mangoes) showed antimicrobial activity against strains previously isolated from mango, detecting significant differences (*P* < 0.05) among treated and control mangoes after 4 and 12 days of storage. *Ps. fluorescens* and *Ps. stutszeri* were the most sensitive.

## Introduction

Mango (*Mangifera indica* L.) is considered one of the world fruits of choice for its unique flavor and attractive appearance, in addition to multiple nutritional properties (Mitra and Baldwin, 1997). Among internationally traded tropical fruits, mango is at the top list in quantity and value. With an annual production of 1,536,654.28 tones (SIAP, 2012a), Mexico is one of the main producers of mango and the second largest exporter. Mexican mango exports and its derivatives have risen 77% over the past seven years (SIAP, 2012b), so the export potential is of great importance.

However, some Mexican fresh products have been involved in food-borne outbreaks in the US, the main export destination (CDC, 2012a). Recently, on September 13, 2012, the Food and Drug Administration (FDA) of The United States (US) warned consumers against eating mangoes from Agricola Daniella, a mango supplier with multiple plantations and a single packing house located in Sinaloa, Mexico (CDC, 2012b). Therefore, it is essential to ensure the safety of this fruit.

Some of the problems related with the safety of mangoes can be directly associated with the postharvest desinfestation treatments, commonly applied to prevent the incidence of agricultural pests such as the fruit fly. As a condition for entry into the US, Mexican mangoes shall receive a Hot Water Immersion Treatment (HWIT) as specified in the APHIS PPQ Treatment Manual (Jacobi et al., 2001; USDA and SAGARPA, 2012). However, this treatment may represent a point of contamination. In fact, it was reported as the cause of an outbreak (Sivapalasingam et al., 2003; Penteado et al., 2004).

UV-C irradiation (190–280 nm) is a new alternative postharvest desinfestation treatment, which represents an interesting application for its germicidal effect and reduction of physiological disorders occurring during cold storage of fruits and vegetables. The UV-C treatment in fruits acts in two ways: directly on the microorganisms on the surface, inactivating them (damaging microbial DNA) and reducing their growth, and indirectly, by inducing the synthesis of compounds related with defense mechanisms and antimicrobial activity, such as phenols, flavonoids, phytoalexins, and polyamines (Marquenie et al., 2003; Rivera Pastrana et al., 2007). On the one hand, Lamikanra and Richard (2004) concluded that low doses of UV-C irradiation stimulate the biosynthesis of phytoalexins, antimicrobial compounds. Specifically in mango, González-Aguilar et al. (2007) reported that UV-C treatments induce accumulation of compounds with antioxidant capacity (polyphenols and flavonoids) in minimally processed mango. On the other hand, Manzocco et al. (2011) demonstrated that UV-C treatment has a high potential for decontamination of “ready to eat” food surface. Yaun et al. (2004) found the reduction of *Salmonella* and *E. coli* O157:H7 on the surface of apples, tomatoes and lettuce with UV-C treatment. Erkan et al. (2001) reported that 10 and 20 min UV-C exposure of slices of zucchini squash, reduced significantly microbial activity and deterioration during subsequent storage at 5 or 10 C. Also, combinations of pulsed white light and UV-C improved the inactivation of conidia of *Botrytis cinerea* and *Monilia fructigena*, responsible for important economical losses during postharvest storage and transport of strawberries and sweet cherries (Marquenie et al., 2003).

The UV-C treatment may also cause significant changes in the natural microbiota of mangoes and the removal of microorganisms having inhibitory action against foodborne pathogens, being that the activities of one microbe may influence the growth and activities of the rest that are present (Schuenzel and Harrison, 2002). Research in microbial ecology and the knowledge of complex interactions among microbes must be better understood to achieve maximal control of pathogens (Doyle et al., 2005). Therefore it is fundamental to study the potential effect of postharvest treatments on the microbial diversity.

On the basis of these considerations, the main objective of this work was to investigate the effect of UV-C irradiation on the bacterial diversity on Ataulfo mangoes surface, using PCR-DGGE analysis of the variable region V3 of 16S rRNA genes. The effect of the treatment on the survival of an *E. coli* isolated from mango was also investigated. Finally, the antimicrobial activity of UV-C treated and non-treated control mango peels on strains isolated previously isolated from mango, and the identification of phenolic compounds, were assessed.

## Materials and methods

### Biological material

Mangoes (Ataulfo variety) at preclimateric state, from Nayarit (Mexico), were used. They were harvested in the period August-October 2005. The fruits were selected according to weight, color, without mechanical or insect damage, to obtain uniform batches and fruits with the same physiological state.

### UV-C treatments

The fruits were placed in plastic trays. Subsequently they were introduced into a UV chamber at a distance of 10 cm from the germicidal UV fluorescent lamp (Sankyo Denky, model 615T8) to be exposed to irradiation for 20 min (250–280 nm). Each fruit was rotated manually to half the exposure time to ensure that the total surface was exposed to the UV light. After this treatment, mangoes were placed in a fume hood for 30 min to remove any ozone produced (González-Aguilar et al., 2004). Immediately they were stored at 25°C and 90–95% relative humidity (RH), for 24 h, in complete darkness, to avoid possible photoreaction effects.

### Sampling for subsequent assays

After storage in complete darkness, the treated fruits were stored at 25°C for 12 days (90–95% RH). In order to evaluate the effect of UV-C treatment on the natural bacterial biota on the surface of mangoes and antimicrobial activity, samples were taken at 0, 4, and 12 storage days. We worked with two batches: 35 fruits with UV-C treatment and 35 non-treated control fruits (samples in triplicate). Each sample was formed by five fruits. The same samples were used for obtaining the mango peel methanol extracts as well as determination of phenolic compounds and antimicrobial activity.

Additionally, we worked with other batches to obtain aerobic mesophilic counts, before and after the UV-C treatment: 40 fruits with treatment and 40 control fruits. Samples were taken at 0, 4, and 12 storage days. Each sample was formed by five fruits.

For the assay to evaluate the survival of *E. coli* on the surface of mangoes with UV-C treatment, we worked with two batches: 66 fruits with UV-C treatment and 66 control fruits (samples in triplicate). The sample size was defined according to recommendations of the International Commission for the Microbiological Specifications for Foods (ICMSF, 2002). For the inoculation, the samples were taken immediately after of storage in complete darkness.

### Effect of UV-C treatment on the natural bacterial biota on the surface of mangoes, determined by 16s rRNA PCR-DGGE

#### Recovery of microbiota on the mangoes surface

The method proposed by Barak et al. (2003) with some modifications was used. Each mango was placed in a sterile zip-lock polyethylene bag (30.5 by 30.5 cm) with 80 mL of sterile deionized water containing 1% Tween 80. The bag was closed and placed in another polyethylene bag. Double-bagged mangoes were placed in an orbital laboratory shaker (RotoMix 50800, Barnstead, Thermoline) and they were agitated for 45 min at 180 rpm. Following agitation, the washing liquid of 5 fruits (one sample) was collected in a sterile glass flask. The obtained mixture was passed through rapid filtration paper to remove dirt and then through a sterile 0.45 μm membrane of nitrocellulose in a Millipore filtration equipment (6-place manifold stainless steel). Filtration finished, the membrane was placed in a sterile centrifuge tube. After 30 min at −20°C, the mixture of microorganisms retained on the membrane was recovered. For this, 2 mL of sterile TES buffer (0.05 M Tris, 0.05 M NaCl, 0.05 M EDTA, pH 8) was added to the centrifuge tube with the frozen membrane. The tube was vigorously agitated for 5 min with a Vortex Mixer 120 V (Fisher Scientific) and the microorganisms were recovered as a homogeneous suspension.

#### DNA extraction

Total DNA was extracted according to routine method based on an enzymatic disruption of cells (lysozyme, pronase, RNase, and SDS) followed by a phenol-chloroform isoamyl alcohol extraction of DNA and a further DNA precipitation with absolute ethanol. For this, the suspension with the recovered microorganisms (2 mL) was transferred to four 1.5 mL microtubes and was homogenized for 10 s at the maximum speed with a Vortex Mixer 120 V (Fisher Scientific). Immediately, 20 μL of a lysozyme solution (10 mg/ml]) were added to each microtube, the samples were gently shaken and incubated at 37°C for 1 h. Then 8 μL of pronase solution (20 mg/ml) and 8 μL de RNAsa (20 mg/ml) were added to each microtube, the samples were gently shaken and incubated at 65°C for 1 h. To continue 120 μL of a sodium dodecyl sulfate solution (SDS, 10% W/V) were added to each microtube, the samples were vortexed for 10 s and incubated again at 65°C for 30 min. After incubation, 600 μL of a mixture of phenol-chloroform-isoamyl alcohol (25:24:1, vol/vol/vol) were added to each microtube and the microtubes were vortexed for 10 s to form an emulsion, after this, they were centrifuged at 5000 rpm for 10 min. Three phases were obtained and each supernatant (containing the DNA) was recovered and transferred to clean microtube. In order to precipitate the DNA, two volumes of absolute ethanol at −20°C were added, the microtubes were vortexed for 10 s and centrifuged at 14,000 rpm for 10 min. Then, the supernatant was removed by decantation and the pellet was allowed to dry at room temperature for 24 h. Finally, the pellet was resuspended in 50 μL of sterile TE buffer (0.01 M Tris, 0.001 M EDTA, pH 8) and incubated at 55°C for 1 h. The contents of the four microtubes representing the same sample were mixed and were stored at −20°C until use. DNA quality was checked on 2% agarose gels (ethidium bromide staining) and the concentration was determined by spectrophotometric estimation. These DNA preparations were used as template in the PCR.

#### Amplification of the V3 region from the 16s rDNA gene by PCR

The amplification of the V3 region from the 16S rDNA gene was carried out using the primers Agc338F (5′-CGCCCGCCGGGCGGCGGGCGGGGCGGGGGCACGGGGGGACTCTACGGAGGCAGCAG-3′) and B518R (5′-ATTACCGCGGCTGCTGG-3′) as described by Muyzer et al. (1993). The Agc338F primer contains the GC clamp (40-nucleotide GC-rich sequence). Each reaction mix (50 μL, final volume) in PCR buffer 1X, contained 75 ng of template DNA, each primer at a concentration of 0.2 μM, each deoxynucleoside triphosphate at a concentration of 0.4 mM, MgCl_2_ at a concentration of 2.5 mM, 1 U of Taq polymerase and bovine serum albumin (BSA) at a concentration of 400 ng/μL. The BSA was added for relief of inhibition from potential PCR inhibitors (Kreader, 1995; Wilson, 1997). All PCR reactions were performed at least in triplicate. The amplification was performed with a Biometra Tpersonal thermal cycler and the protocol of amplification used is described by Ampe et al. (1999) (Table 1). A “touchdown PCR” was performed to reduce the formation of spurious by-products. For this, the initial annealing temperature used was 10°C above the expected annealing temperature (65°C), and the temperature was decreased by 1°C every second cycle until the touchdown temperature, 55°C, was reached; then 10 additional cycles were carried out 55°C. The quality of the amplicons obtained (233 bp) was analyzed by electrophoresis on 2% agarose gel (ethidium bromide staining) prior to DGGE.

**Table 1:** **Protocol of amplification of the V3 region from the 16S rDNA using the primers Agc338F and B518R**.

**Phase**	**Cycles**	**Temperature (°C)**	**Time (min)**
Denaturing	1	94	5
Denaturing		94	1
Annealing	20	65–55	1
Extension		72	3
Denaturing		94	1
Annealing	10	55	1
Extension		72	3
Extension	1	72	10

#### Denaturing gradient gel electrophoresis (DGGE)

DGGE was performed using the DCode Universal Mutation Detection System (Bio-Rad Laboratories) as described in the instructions of the manufacturer. Polyacrylamide gels (8% of a 37:1 acrylamide/bisacrylamide mixture in 1X TAE buffer), with a gradient of 15 to 60% denaturant (100% corresponds to 7 M urea and 40% formamide w/V), were made with a gradient maker (Bio-Rad Laboratories). Equal volumes of PCR reactions (40 μL) were loaded on gels, and run for 17 h at 85 V in TAE buffer (0.04 M Tris, 0.02 M acetic glacial acid, 0.001 M EDTA, pH 8) at a constant temperature of 65°C. The running conditions were optimized previously. Likewise, perpendicular DGGE was performed before to determinate the optimum gradient (60°C, 130 V, 2 h). After electrophoresis, the gels were stained with the DNA silver kit “Plus One DNA” (Pharmacia Biotech) and photographed with a Fluor-S system (BioRad).

DGGE separation of different PCR reactions was performed in several replicates to check reproducibility.

#### Recovery of DGGE fragments (individual bands excision), sequencing and analysis of the sequence data

The most interesting bands were selected and cut individually with a sterile scalpel. To recover the DNA in each band, the method described by Gafan and Spratt (2005) was used. Each band excised was placed in a microtube containing 50 μL of sterile deionized water and DNA was eluted through incubation at 37°C for 1 h and passive diffusion (it was stored at 4°C for 24 h). This eluted DNA was used as template for a further PCR. Ten μL were added to the reaction mix (50 μL, final volume), maintaining the reagent concentrations specified above and the same amplification protocol. In this case, the amplification was carried out using the primers 338F (5′-ACTCCTACGGGAGGCAGCAG-3′) and B518R (5′-ATTACCGCGGCTGCTGG-3′). The PCR amplicons were purified with PCR Cleanup kit (Qiagen, Mississauga, ON, Canada) as described in the instructions of the manufacturer. The success of this procedure was checked by electrophoresis on 2% agarose gel (ethidium bromide staining). Finally, the purified PCR products were sent to sequencing with primer B518R (see above) at the Institute of Cell Physiology, UNAM.

Basical Local Aligment Search Tool (BLAST), available at GenBank, was used. We consider that the sequences with 90% or more identity correspond to the same species (Chakravorty et al., 2007).

### Effect of UV-C treatment on the aerobic mesophilic microorganisms on the surface of mangoes, determined by plate count

As DGGE was done with DNA extraction only, it is not possible to obtain information on survival of bacteria. For this, aerobic mesophilic counts were done before and after the UV-C treatment. Samples were taken as specified at the Sampling for Subsequent Assays section. Recovery of microbiota on the mangoes surface was performed as specified at the Recovery of Microbiota on the Mangoes Surface section. From the suspension with the recovered microorganisms (2 mL), decimal dilutions were prepared with 0.1% bacteriological peptone; 0.1 mL samples were inoculated into nutrient agar plates. They were incubated at 37°C for 48 h and plates with 15–45 CFU were counted. Results were expressed as CFU/mL.

### Survival of *E. coli* on the surface of mangoes with UV-C treatment

An ampicillin resistant *E. coli* strain (109289-B) previously isolated from the surface of mango and identified as EPEC (enteropathogenic *E. coli*) was used for this assay. The strain was maintained in nutrient broth with 20% glycerol at −70°C.

#### Inoculum preparation and inoculation of the fruits

The EPEC strain was reactivated in brain heart infusion (BHI) at 37°C for 24 h and subcultured on nutrient agar with 50 μg ampicillin. The plates were incubated at 37° C for 24 h. One colony was subcultured into BHI and incubated at 37°C for 16 h. To prepare the inoculum, the microorganism suspension was centrifuged at 15,000 rpm for 15 min at 4°C. The supernatant was discarded and the pellet resuspended in 5 mL 0.1% peptone water. This procedure was repeated.

The fruits sampled as specified at the Sampling for Subsequent Assays section, were inoculated under aseptic conditions at ambient temperature in the following way: 100 μL inoculum (1.9 × 10^8^ CFU/mL) was placed as small drops on each fruit in previously marked areas and left to dry during 1 h at ambient temperature. The fruits were then stored at 25°C during 8 days (RH 90–95%).

#### Microbiological analyses

Each marked area was swabbed with a cotton swab previously wet with 0.1% peptone. It was then placed in a vial with the same diluent, stirred for 1 min. Serial dilutions were performed and plated on nutrient agar-ampicillin. The plates were incubated at 37°C for 24 h. CFUs were determined. Samples were taken by triplicate at 0, 2, 4, 8, 24, 48, 72, 96, 120, 144, 168, and 192 h after inoculation. As negative control the same area on the fruits without inoculation and swabbed in the same way was used. As positive control, 100 μL inoculum were diluted in 5 mL 0.1% peptone water and this was plated as above. Results were expressed as CFU/mL.

### Extraction and determination of total phenolic compounds

Extracts were prepared according to the method proposed by Ajila et al. (2007). For this, 20 g of mango peel was weighed and crushed in a mortar using liquid nitrogen. Immediately, 100 ml of methanol at 80% (V/V) was added and shaken for 1 min. It was kept in a bath at 70°C for 2 h and then it was passed through a Whatman filter paper no. 4. Finally, the total phenolics were assayed colorimetrically by the Folin-Ciocalteu method, according to the protocol suggested by Slinkard and Singleton (1977). For this, 1500 μL of distilled water, 100 μl of Folin-Ciocalteu reagent, and 200 μl of phenolic extract were mixed. After 5 min 200 μL of 20% Na_2_CO_3_ was added and the mixed was shaken at constant speed. The absorbance was measured at 765 nm (Termo Spectronic model Genesis 10 UV spectrophotometer) after 30 min at room temperature. A mixture of water and reagents was used as a blank. Total phenolic content was expressed as mg/g fresh tissue.

### Determination of antimicrobial activity of mango peel methanol extracts

The antimicrobial activity was determined against 14 Gram-negative (GN) bacterial strains: *Pseudomonas fluorescens* M1, *Ps. stutzeri* M2, *Ps. aeruginosa* M3, *Enterobacter cancerogenus* M4, *E. hormanechei* M5, *E. cloacae* M6, *E. aerogenes* M7, *Klebsiella pneumonia* M8, *K. ornithinolytica* M9, *Citrobacter freundii* M10, *C. amalonatus* M11, *Serratia marcescens* M12, *Escherichia coli* 109289-B, *Salmonella* sp. They belong to collection of strains previously isolated from mangoes and were maintained in nutrient broth with 20% glycerol at −70°C. The strains were activated in nutrient broth at 37°C for 24 h. Immediately they were plated on nutrient agar in 9-cm-diameter Petri dishes, incubated at 37°C for 24 h. After this time, a colony was selected and placed in nutrient broth, incubating at 37°C for 24 h. Then, the concentration of each microorganism was adjusted to 0.5 tube of Mc Farland scale (1 × 108 UFC/mL).

The antimicrobial activity of phenolic compounds was carried out using the agar diffusion method (Balakrishnan et al., 2006). This test was performed on sterile filter paper disks (6 mm diameter). For each strain, the following procedure was carried out:

The microorganisms suspended in nutrient broth were inoculated on nutrient agar in 9-cm-diameter Petri dishes. Afterward, four filter paper disks were impregnated with 50 μ L from phenolic extracts. The disks were placed on the plates inoculated with the GN bacterial strain. The plates were incubated at 37°C for 24 h. After this period, visual readings were performed by observing the presence of a bacterial growth inhibition zone. The halo of inhibition zone was measured in millimeters, with the aid of a millimeter ruler. As a negative control, one of the disks was impregnated with 80% methanol (V/V). As a positive control, other disk was impregnated with 1% acetic acid (V/V).

### Identification and quantification of phenolic compounds by high pressure liquid chromatography (HPLC)

In order to identify the individual phenolic compounds, a HPLC assay was carried out according to the method proposed by Singh et al. (2004). Phenolic extracts were analyzed on 1525 Waters HPLC System (Waters, Milford, MA) with binary pumps attached to Symmetry C 18 column (5 μm particle, 3.9 × 150 mm, Waters, Milford, MA). The samples were applied to C 18 column and eluted over a gradient of solvent A:B (20:80, 40:60, 80:20, and 20:80) at a flow of 1 mL/min at room temperature; 20 μL of sample was injected. The mobile phase consisted of (A) Methanol and (B) 0.4% (V/V) Acetic Acid. The phenolic compounds were monitored at 290 nm in a Waters 2487 Dual Wavelength Absorbance Detector (Waters, Milford, MA).

As standards, gallic acid, caffeic acid, chlorogenic acid, ferulic acid, cinnamic acid, and coumaric acid were used. Identification of the unknown phenolics was based on matching their retention times with those of pure standards of phenolics. Peak area was used for quantification using external standard calibration curves. For this, the software Breeze (Waters, Milford, MA) was used.

### Statistical analysis

All experiments were performed on triplicate and the experimental data were reported as average and provided with Standard Derivation (SD). Statistical ANOVA and Duncan test (*P* ≤ 0.05) were performed using SPSS (Statistical Package for the Social Sciences version 14.0, Student). Statistical ANOVA was carried out to evaluate the effect of UV-C treatment on the survival of *E. coli* and the phenolic compounds levels.

## Results

### Effect of UV-C treatment on the natural bacterial biota on the surface of mangoes

**Figure 1:**
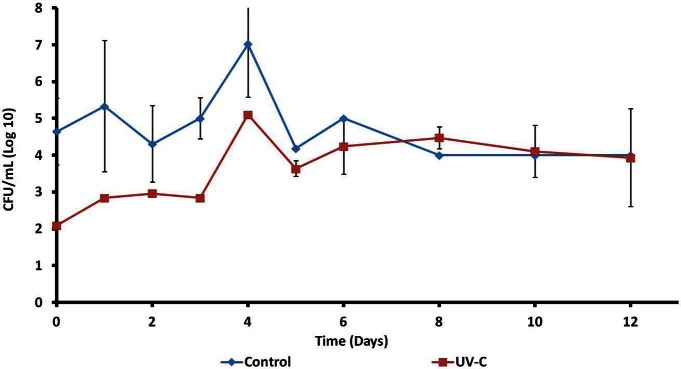
**Effect of UV-C treatment on the aerobic mesophilic microorganisms on the surface of mangoes**.

In accordance with the assay to determine the effect of UV-C treatment on the total viable count, described at Effect of UV-C Treatment on the Aerobic Mesophilic Microorganisms on the Surface of Mangoes, Determined by Plate Count section, the UV-C treatment reduce the microbial load on the surface of mangoes, immediately after treatment. However the microbial load increases during storage, in both control and treated samples. The largest aerobic mesophilic counts were obtained on the day 4. Subsequently, these counts decreased slightly and were kept constant until day 12. At this point, no significant differences were found between treated and control fruits. The results are shown in Figure 1.

**Figure 2:**
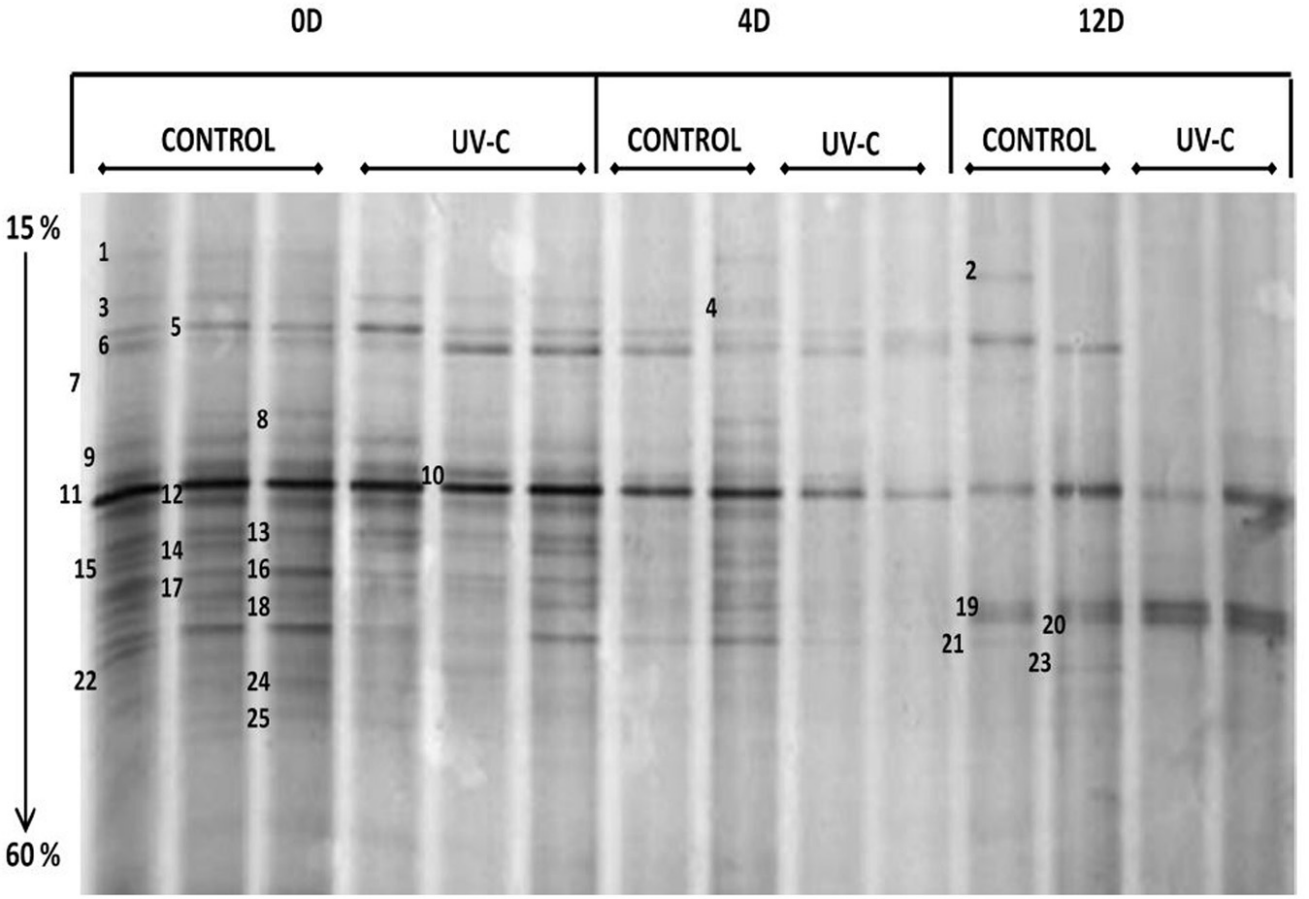
**DGGE band patterns obtained from samples of bacterial microbiota on the surface of mangoes with and without UV-C treatment.** 0D, immediately after treatment; 4D, 4 days after treatment; 12D, 12 days after treatment. Denaturing gradient: 15 to 60%. Electrophoresis conditions: 85 V, 17 h, 65°C.

On the other hand, the DGGE band patterns obtained are shown in Figure 2. The reproducibility of the results was verified by the band patterns similarity from the same batch.

It was possible to detect clear differences between the band patterns obtained for control, treated, and stored mangoes, so that PCR-DGGE was a convenient and rapid method to determine the structure of natural bacterial biota on the surface of mangoes and the effect of UV-C treatment, as well as to detect the modifications on the bacterial composition during storage.

Time zero (just after UV-C treatment), mangoes showed complex patterns of approximately 25 bands. Control and treated fruits share most of the bands (Figure 1), although with some differences regarding to intensity (relative abundance). This result was confirmed by the assay to determine the total viable microbiota immediately after UV-C treatment. The microbial load on the surface of mangoes was reduced approximately 2.6 logarithms.

Results of band identification by 16S rRNA gene (V3 region) sequence comparison (Table 2) were not a species per band, but a list of several species or genera for which the same percent of similarity was obtained with the BLAST program, possibly because of the small size of the amplicons (233 bp). Even when it was not possible to have a full identification, in most cases it includes members of a genus or of a family. Twelve genera were identified on the surface of mangoes, predominating *Enterobacter, Pantoea*, and *Klebsiella*. In a smaller proportion, *Escherichia, Erwinia, Salmonella, Lactococcus, Weissella, Lactobacillus, Citrobacter, Pseudomonas*, and *Bacillus* genera were identified.

**Table 2:** **Identification of bacterial species isolated from mango**.

**Band number**	**Identification**	**Percent identity**
1	NI	-
2	NI	-
4	*Pantoea* sp., *P. ananatis, Enterobacter sakazakii, Escherichia coli, Pantoea agglomerans, Klebsiella* sp.	97
5	*Enterobacter sakazakii, Pantoea* sp., *Enterobacter* sp., *E. coli, Bacillus* sp.	97
8	*Lactococcus lactis*	96
10	*Enterobacter* sp., *Pantoea* sp., *P. dispersa, P. agglomerans, P. stewartii, Erwinia* sp., *E. stewartii*	96
11	*Pantoea agglomerans, P. dispersa, Erwinia* sp., *Enterobacter cloacae, Escherichia hermannii, E. coli*	95
12	*Enterobacter* sp., *E. pyrinus, E. cloacae, E. asburiae, E. agglomerans, E. hormaechei, E. hermannii, Klebsiella* sp., *Salmonella enterica subsp*., Arizonae	94
13	*Enterobacter* sp., *E. hormaechei, Klebsiella oxytoca, K. pneumoniae*	99
14	*Citrobacter* sp., *C. farmeri, Citrobacter amalonaticus, Enterobacter cloacae, Salmonella* sp., *S. enterica subsp., Indica*	95
15	*Enterobacter* sp., *E. cloacae, Pantoea agglomerans, K. oxytoca*	98
19	*Weissella* sp., *W. confusa, W. cibaria, Lactobacillus* sp., *L. confusus, L. viridescens*	90
20	*Klebsiella* sp., *K. pneumonia, K. oxytoca, Enterobacter asburiae, E. aerogenes*	99
21	*Pseudomonas* sp., *P. putida, P. fluorescens*	90
22	*Pantoea* sp.	98
24	NI	-
25	NI	-

NI, not identified.

Regarding the monitoring of samples during storage, the number of bands decreased progressively in both control and treated samples. Most differences were shown at day 4. On the 12 day, control mangoes showed patterns of less than 10 bands and treated fruits showed only 2–4 bands. Note that although the decrease of bacterial diversity was detected, the microbial load increases during storage.

The storage had an important effect on genera only presents in control samples (untreated fruits). Bands 8 (*Lactococcus lactis*) and 4 (*Pantoea* sp., *P. ananatis, Enterobacter sakazakii, Escherichia coli, Pantoea agglomerans, Klebsiella* sp.) were detected up to 4 days and then disappeared at 12 days, while bands 15 (*Enterobacter* sp., *E. cloacae, P. agglomerans, K. oxytoca*) and 22 (*Pantoea* sp.) were only detected at day zero.

Bands 5, 10, 11, 12, 19, 20, and 21 were detected in treated and control samples.

Band 11, identified as *Pantoea agglomerans, P. dispersa, Erwinia* sp., *Enterobacter cloacae, Escherichia hermannii*, and *E. coli*, is present during the whole storage period, in both control and treated samples. It is the only band that is present throughout the ripening in all samples.

Bands **5** (*Enterobacter (Cronobacter) sakazakii, Enterobacter* sp., *Pantoea* sp., *E. coli* and *Bacillus* sp.), **12** (*Enterobacter* sp., *E. pyrinus, E. cloacae, E. asburiae, E. agglomerans, E. hormaechei, E. hermannii, Klebsiella* sp., *Salmonella enterica* subsp. Arizonae), **19** (*Lactococcus* sp., *Lactobacillus* sp., and *Weissella* sp.) and **21** (*Pseudomonas* sp.) were present in control samples during the whole storage period. However, in treated mangoes, bands 5 and 12 were detected up to 4 days and then disappeared at 12 days, band 21 was only detected at day zero and band 19 was detected on the day 12.

Band 10 (*Enterobacter* sp., *Pantoea* sp., *P. dispersa, P. agglomerans, P. stewartii*) was detected until day 4 of storage and then disappears, in both control and treated samples.

Band 20 (*Klebsiella* sp., *K. pneumoniae, K. oxytoca, Enterobacter absuriae, E. aerogenes*) was present in control samples at day zero and day 12, but not at day 4. In treated fruits, this band was detected on the 12 day.

### Survival of *E. coli* on the surface of mangoes with and without UV-C treatment

**Figure 3:**
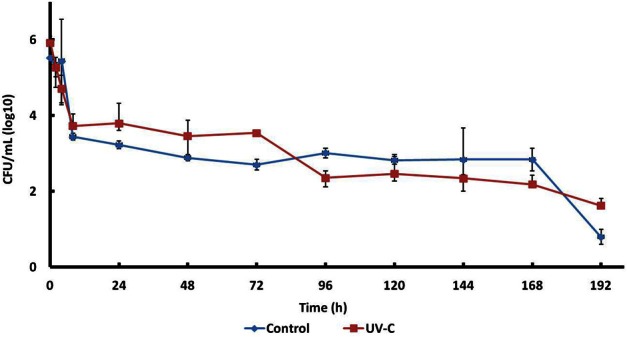
**Survival of *E. coli* 109289-B on the surface of mangoes with or without UV-C treatment**.

Survival of an *E. coli* strain (isolated previously from mango) inoculated on the surface of control and UV-C treated mangoes stored at 25°C was studied (Figure 3). This strain survived 8 days on the surface of fruits (treated and control samples).

*E. coli* counts both in treated and control mangoes were reduced approximately 2.5 logarithms in the first hours of storage, then slowly to 2.0–2.5 log CFU/mL at 168 h, and to 0.5–1.5 log CFU/mL at 192 h.

Growth of *E. coli* on treated and control mangoes was not significantly different.

### Phenolic compounds and antimicrobial activity of treated and control mango peel methanol extracts

Although higher concentration of total phenols in non-treated control mango peels (375 mg/g) was found, the phenolic content in UV-C treated and control mango peels, was not significantly different. At 12 days of storage, the concentration of phenols in UV-C treated and control was very similar.

HPLC analyses showed the presence of chlorogenic, gallic, and caffeic acids in mango peels. Significantly higher concentrations of chlorogenic and gallic acids (but not of caffeic acid) were found in treated as compared to untreated fruits. Besides, the concentration of these compounds increased with storage time at 25°C.

Mango peel methanol extracts of control (untreated) and UV-C treated fruits showed antimicrobial activity against strains isolated from mango. Significant differences (*P* < 0.05) in antimicrobial activity among untreated and treated mangoes after 4 and 12 days were detected for methanol extracts (Table 3).

**Table 3:** **Antimicrobial activity of methanol extracts prepared with mango peels with or without UV-C treatment on bacterial strains isolated from mango**.

**Bacteria**	**Storage time (days)**	**Diameter of inhibition (mm)**	
		**Control**	**Treated**	
*Ps. fluorescens* M1	0	**36**	**35**
	4	**31**	**45**
	12	**37**	**45**
*Ps. sutzeri* M2	0	**30**	**40**
	4	**32**	**40**
	12	**35**	**35**
*Ps. Aeruginosa* M3	0	ND	**22**
	4	ND	**22**
	12	ND	**16**
*E. cancerogenus* M4	0	ND	ND
	4	ND	ND
	12	ND	ND
*E. hormanechei* M5	0	**22**	**24**
	4	ND	**23**
	12	ND	**25**
*E. cloacae* M6	0	ND	ND
	4	ND	**18**
	12	ND	ND
*E. aerogenes* M7	0	ND	ND
	4	ND	**18**
	12	ND	ND
*K. pneumoniae* M8	0	ND	ND
	4	ND	ND
	12	ND	ND
*K. ornithinolytica* M9	0	ND	ND
	4	ND	16
	12	ND	18
*C. freundii* M10	0	ND	ND
	4	ND	16
	12	ND	20
*C. amalonatus* M11	0	ND	ND
	4	ND	ND
	12	ND	ND
*S. marcescens* M12	0	ND	ND
	4	ND	ND
	12	ND	ND
*Salmonella sp*.	0	ND	ND
	4	ND	12
	12	ND	15
*E. coli* 109289-B	0	ND	ND
	4	ND	15
	12	ND	18

ND, not detected. The diameter of inhibition of most sensitive strains were highlighted in bold.

*Ps. fluorescens* M1 and *Ps. stutszeri* M2 were the most sensitive; however *Ps. aeruginosa* M3, *E. hormanechei* M5, *E. cloacae* M6, and *E. aerogenes* M7 were more susceptible to treated mango extracts after 12 days, compared to those of treated fruit extracts after day 4. Chlorogenic acid was detected with the highest concentration in those extracts. The rest of strains were more sensitive to the acetone extract (data not shown), in which gallic acid was found with the highest concentration. The highest values of antimicrobial activity were found for treated fruits acetone extracts, as they contained 30% higher gallic acid concentrations compared to untreated mangoes, after 4 days storage (data not shown).

## Discussion

### Effect of UV-C treatment on the natural bacterial biota on the surface of mangoes

With hydrothermal treatment, contamination of mangoes during cooling in water represents a risk. In December 1999, the US Centers for Disease Control and Prevention (CDC) detected an outbreak due to *Salmonella enterica* serotype Newport (SN) linked to the consumption of imported mangoes. Traceback of the implicated mangoes led to a single Brazilian farm, where the cold water immersion after the hot water treatment was identified as a possible point of contamination (Sivapalasingam et al., 2003; Penteado et al., 2004).

UV-C irradiation is an interesting alternative, but like all postharvest treatments, it may also cause significant changes in the natural microbiota of mangoes.

The results of this study, suggest that this treatment did not have a significant effect immediately after UV-C irradiation on the bacterial diversity, only on the relative abundance. The microbial load on the surface of mangoes was reduced approximately 2.6 logarithms; similar results were obtained by Manzocco et al. (2011) in irradiated fresh-cut melon. Genera as *Lactococcus* and *Pantoea* only detected on the surface of control mangoes and disappear with the treatment. This suggests that these genera were affected by the UV-C treatment.

Most modifications of the bacterial composition on the surface of mangoes (control and treated samples) occurred during storage at 25°C (4 and 12 days), mainly at day 4. It is possible that at this moment, important biochemical changes (as pH changes) are occurring due to the ripening process. Compared to control, UV-C treated mangoes at 4 days showed important modifications in the band patterns, as all but bands 6 and 11 decreased intensity. This indicates the effect of UV-C treatment during storage. After 12 days, both control and treated mangoes patterns were modified, treated fruits show 3 intense bands (11, 19, and 20), which are also present in the control, and control contain additionally bands 2, 5, 21, and 23. Modifications in the non-treated control mangoes during storage suggest factors different from UV-C treatment have occurred.

Twelve genera were identified from control and treated zero time DGGE sequenced band patterns. GN predominating (75%) and *Enterobacter* spp. representing 50% of the total bands. Our results agree with several studies that have found *Enterobacter* genera dominating fresh vegetables. Schuenzel and Harrison (2002) showed that 92% of the bacterial strains isolated from the natural microbiota of vegetable products, were GN. Oliveira et al. (2012) reported that vegetal products are usually colonized by a great variety of microorganisms, generally GN bacteria, members of *Pseudomonadaceae* and *Enterobacteriaceae* families. Moreover, Randazzo et al. (2009), found members of the *Enterobacteriaceae* family dominating fresh vegetables, with the plant pathogens *Erwinia, Enterobacter*, and *Pantoea*. These genera were also found in this work.

*Enterobacter* has also been reported as human pathogen (Harris et al., 2003), however, *E. asburiae* is reported as a competitor strain, because of its ability to use efficiently the same carbon and nitrogen compounds as *E. coli* O157:H7 (Cooley et al., 2006).

Band 11 (*Pantoea agglomerans, P. dispersa, Erwinia* sp., *Enterobacter cloacae, Escherichia hermannii, E. coli*) is present during the whole storage period, in both control and treated samples. This is important, because *P. agglomerans* has been reported as antagonist of *Penicillium digitatum* in lemons (Plaza et al., 2004), but also agglomerating with *Salmonella* on cilantro surface, increasing its survival up to 100 days (Aruscavage et al., 2006).

Lactic acid bacteria were found principally in control samples. This suggests that they would be important members of the natural microbiota and act as antagonists, due to the production of acids and possibly, bacteriocins. In fact, Parish et al. (2003) and Liao and Fett (2001) have reported that lactic acid bacteria are capable of avoiding excessive growth of pathogens on the surface of vegetal products.

On the other hand, *Pseudomonas* was present throughout storage time in control and on day zero in treated samples. Martins et al. (2012) reported that in grapes, *Pseudomonas* counts decrease when the fruit is mature. Members of this genus are typically found in soil and plants. They have also been isolated from vegetables used to prepare salads. It usually produces exopolysaccharides, which could be used to make biofilms. They are associated to plant matrixes and are not easily eliminated during washing procedures. Also, *Ps. fluorescens* has been reported as opportunistic pathogen (Randazzo et al., 2009).

### Survival of *E. coli* on the surface of mangoes with and without UV-C treatment

Fruits and vegetables consumption is considered a risk factor due to the possible presence of enterobacterial pathogens, mainly *Salmonella* and *E. coli*, then a better knowledge of their ecology in these products is essential to propose control measures.

No significant differences were observed in the growth of *E. coli* on treated and control mangoes. In both cases counts decreased rapidly during the first hours and then slowly, so that it was not eliminated after 192 h. The strain had been isolated from mango, so this is possibly the reason for its survival. The strain could have been adapted to persist on the surface of mango.

It is clear that the changes in microbial ecology after UV-C treatment and during storage, did not affect the survival of the EPEC strain in this study. However, it is important to consider that some authors have reported that some bacterial genera identified in this work (members of the natural biota on the surface of mango) present antagonistic activity against foodborne pathogens.

Babic et al. (1997) showed that the genus *Pseudomonas* predominates on plant products and has the ability to limit the growth of *L. monocytogenes* and *E. coli* O157:H7. Schuenzel and Harrison (2002) obtained isolates from the natural biota of vegetables, as carrots, lettuce, and cilantro and found among them isolates capable of inhibiting the growth of *E. coli* O157:H7, *L. monocytogenes, Salmonella Montevideo*, and *Staphylococcus*. Also, they showed that strains of *Pseudomonas fluorescens* have antagonic effects on *L. monocytogenes*. Johnston et al. (2009) isolated *Pantoea, Pseudomonas, Klebsiella, Enterobacter, Aeromonas, Burkholderia*, and *Serratia* that showed inhibitory activity against *E. coli* O157:H7. Approximately 17% of these genera produce antimicrobial peptides and 16% produce acids, as part of their antimicrobial activity. Critzer and Doyle (2010), isolated members of the genera *Pantoea, Pseudomonas, Klebsiella, Enterobacter, Aeromonas, and Burkholderia*, that had the ability to inhibit *E. coli* O157:H7 on lettuce and spinach leaves. Cooley et al. (2006) reported that *Enterobacter asburiae* is a competitor strain, because of its ability to use efficiently the same carbon and nitrogen compounds as *E. coli* O157:H7.

Interactions of resident enterobacteria can be benefic, harmful, or neutral (Aruscavage et al., 2006). Some moulds as *Cladosporium* sp., may promote the growth of enterobacteria, as they increase the pH value around them (Brandl, 2006).

Competition for nutrients could be the mechanism that this bacteria uses. This is a little information about the interaction between human pathogens and the natural microbiota on plants and vegetables products, which could contribute to the suppression of human pathogens.

### Phenolic compounds and antimicrobial activity of treated and control mango peel methanol extracts

No significant differences were detected in the concentration of total phenols among treated and control mango peels, however, at day 4, we detected differences in the type of phenolic compounds presents, in treated and control mangoes. The highest values of antimicrobial activity were found for treated fruits acetone extracts, as they contained 30% higher gallic acid concentrations compared to control mangoes.

Therefore, it is possible that on day 4, the gallic acid is likely to play an important role in the antimicrobial activity in treated fruits. This suggests an effect of the UV-C irradiation in the synthesis of phenolic compounds.

It has been found that gallic acid and its derivatives are active against Gram-positive and GN bacteria (Binutu and Cordell, 2000).

In the band patterns obtained by DGGE (Figure 2), we observed that certain bands were present in control samples during the whole storage period, however, in treated mangoes these bands were only detected up to 4 days. Possibly these bands correspond to species that are sensitive to certain phenolics compounds as gallic acid. In fact, band 21 (*Pseudomonas* sp.) was only detected at day zero in treated fruits. This is important because *Ps. fluorescens* and *Ps. stutszeri* were the strains most sensitive in the assay to determine the antimicrobial activity of phenolic extracts.

The mechanism of action of phenolic compounds has not been completely dilucidated, but it is known that terpenols and phenols disrupt membranes and tannins denature proteins and death can be caused by degradation of the cell wall, damage to cytoplasmic membrane and membrane proteins, filtration of intracellular content and cytoplasm coagulation (Negi, 2012). Different phenolic acids exert different antimicrobial activity, and this activity depends on environmental factors, as pH, water activity and the microorganism (Gómez et al., 2010).

## Conclusions

It was possible, through the use of PCR-DGGE, to detect modifications on the bacterial composition of the surface; it is a convenient and rapid method. Principal modifications of the bacterial composition on the surface of mangoes treated with UV-C occurred during storage (4 and 12 days); this effect was not observed immediately after treatment. These modifications did not affect survival of *E. coli* on the surface of mango, however, genera that have been recognized as antagonists against foodborne pathogens, were identified in the band patterns. Chlorogenic, gallic, and caffeic acids were identified in mango peels. Significant differences were detected in chlorogenic and gallic acids concentrations among treated and non-treated mangoes. Phenol extracts of treated and control fruits showed antimicrobial activity against bacterial strains isolated from mango. *Ps. fluorescens* and *Ps. stutszeri* were the most sensitive Our results suggest that phenolic compounds play an important role to limit the survival of enterobacteria on the fruits.

### Conflict of interest statement

The authors declare that the research was conducted in the absence of any commercial or financial relationships that could be construed as a potential conflict of interest.
